# Supplementary material for: Super-forecasting the ‘technological singularity’ risks from artificial intelligence
Source: Evol Syst (Berl). 2022 Jun 4;13(5):747–57. doi: 10.1007/s12530-022-09431-7 (PMC9166151; doi:10.1007/s12530-022-09431-7)
Supplement: Supplementary file 1 — Supplementary Material 1 [file 12530_2022_9431_MOESM1_ESM.docx]

Response to reviewers

Reviewer #1: 1. The section 5 named "Results and Forecasts" given some current AI cyber-risk solutions, AI's future role forecasts in the cybersecurity, and cybersecurity solutions based on AI algorithms. The forecasts were all-inclusive.

- we have included a clarification in the titles of where the results are presented in relation to the forecasts. We also listed 6 solutions in Table 4, with specific solutions.

2. Some explanations of figure 3 should be added to help readers to understand the implications. Moreover, what is the difference between "build algorithms" and "develop algorithms" in this figure?

- We added a new paragraph after figure 3, explain the rationale between ‘build’ and ‘develop’ wordings.
